# Feasibility and Safety of Chronic Total Occlusion Percutaneous Coronary Intervention via Distal Transradial Access

**DOI:** 10.3389/fcvm.2021.673858

**Published:** 2021-05-10

**Authors:** Cheng-Jui Lin, Wei-Chieh Lee, Chieh-Ho Lee, Wen-Jung Chung, Shu-Kai Hsueh, Chien-Jen Chen, Cheng-Hsu Yang, Hsiu-Yu Fang, Cheng-I Cheng, Chiung-Jen Wu

**Affiliations:** Division of Cardiology, Department of Internal Medicine, Kaohsiung Chang Gung Memorial Hospital, Chang Gung University College of Medicine, Kaohsiung, Taiwan

**Keywords:** chronic total occlusion, percutaneous coronary intervention, distal transradial access, snuffbox access, vascular complication

## Abstract

**Aims:** The current study aims to verify the feasibility and safety of chronic total occlusion (CTO)-percutaneous coronary intervention (PCI) via the distal transradial access (dTRA).

**Methods:** Between April 2017 and December 2019, 298 patients who underwent CTO PCI via dTRA were enrolled in this study. The baseline demographic and procedural characteristics were listed and compared between groups. The incidences of access-site vascular complications and procedural complications and mortality were recorded.

**Results:** The mean J-CTO (Japanese chronic total occlusion) score was 2.6 ± 0.9 points. The mean access time was 4.6 ± 2.9 min, and the mean procedure time was 115.9 ± 55.6 min. Left radial snuffbox access was performed successfully in 286 patients (96.5%), and right radial snuffbox access was performed successfully in 133 patients (97.7%). Bilateral radial snuffbox access was performed in 107 patients (35.9%). 400 dTRA (95.5%) received glidesheath for CTO intervention. Two patients (0.7%) developed severe access-site vascular complications. None of the patients experienced severe radial artery spasm and only 2 patients (0.5%) developed radial artery occlusion during the follow-up period. The overall procedural success rate was 93.5%. The procedural success rate was 96.5% in patients with antegrade approach and 87.7% in patients with retrograde approach.

**Conclusions:** It is both safe and feasible to use dTRA plus Glidesheath for complex CTO intervention. The incidences of procedure-related complications and severe access-site vascular complications, and distal radial artery occlusion were low.

## Introduction

Transradial access (TRA) is preferred for percutaneous coronary intervention (PCI) because of advantages including less associated access vascular complications, comfort and convenience, early ambulation, and short hospital stays when compared with transfemoral access (TFA) (1–4). For emergency and complex PCI for chronic total occlusion (CTO), TRA has a similar success rate, better safety, and even contributes to better outcomes (5–7).

In the recent years, several interventionists focused on dTRA for routine PCI, and reported a shorter time to hemostasis and a similar procedural success rate to conventional TRA (8–10). However, few reports have assessed the impact of dTRA on complex CTO-PCI. Therefore, we aimed to explore the feasibility and acceptability of dTRA in the anatomical snuffbox for CTO-PCI.

## Materials and Methods

### Study Population

Between April 2017 and December 2019, 298 patients underwent PCI for CTO lesions via dTRA by one experienced operator in our institution. During the same period, a total 479 patients underwent PCI for CTO in our institution and dTRA approach for selective patients was estimated 62.2% in CTO intervention. The retrospective study protocol for dTRA CTO PCI registry was approved by our Institutional Review Board of Kaohsiung Chang Gung Memorial Hospital and was approved by the ethical guidelines of the 1975 Declaration of Helsinki.

### Definitions

Chronic total occlusion lesions were defined according to the thrombolysis in myocardial infarction grade (TIMI) of 0 for more than 3 months. The duration of occlusion was estimated based on the history of angina, history of myocardial infarction (MI) in the same territory, or previous coronary angiography. The definition of procedural success was successful guidewire and balloon crossed with or without final stenting, with residual diameter stenosis ≤ 30%, and the presence of a final TIMI 3 flow without major periprocedural complications (8). The technical success rate was defined as the successful recanalization of CTO lesions. Major adverse cardiovascular and cerebral events (MACCE) were defined as non-fatal MI, acute stent thrombosis requiring repeat revascularization, stroke or transient ischemic attack, and cardiovascular mortality. Periprocedural MI was defined as an increase in troponin-I to >5-fold of the upper reference level (0.3 ng/mL) during the first 48 h following PCI (9). Acute kidney injury was defined as a rise in creatinine of ≥50% from its baseline value and/or a fall in the glomerular filtration rate (GFR) by ≥25% (10).

### Patient Position and Preparation

A position for the patient was described in previous studies (11, 12). The left upper arm was placed comfortably on the left side of the patient after the puncture. The left hand was bent over toward the patient's right groin, while the right hand was placed on the right side of the patient. The operator was positioned near the patient's head for subcutaneous injection. To bring the artery to the surface of the fossa, the patient was asked to grasp his thumb under the other four fingers, with the hand slightly abducted. The artery was punctured, preferably with a 20-gauge open needle, at an angle of 30–45 degrees, and from lateral to medial. The needle was directed to the point of the strongest pulse in the anatomical snuffbox. Most CTO intervention was performed under local anesthesia alone and may change to under conscious sedation if the patients could not tolerate prolonged procedure and could not cooperate well.

After successful puncture, a flexible, soft, J-shaped 0.025" hydrophilic wire (Terumo Corporation, Japan) was inserted, and followed by introduction of the 5-, 6-,7-, or 7-F Glidesheath (Terumo Corporation, Japan). A spasmolytic cocktail containing 200 μg of nitroglycerine and a weight-adjusted dose of heparin was administered through the sheath side port. Bilateral dTRA approach for CTO PCI was demonstrated as Supplementary Figure 1.

First, a 6-F Ikari left 4 or 3.5 guiding catheter (Terumo Corporation, Japan) was used for diagnostic coronary angiography. Guiding catheters including 6F or 7F EBU for left coronary artery and 6F or 7F AL1 for right coronary artery were selected for the antegrade or retrograde approach according to each patient. We used standard guiding catheter first and changed extended guiding catheter if body height of the patient was >180 cm. An intracoronary bolus of heparin was administered via the guiding catheters prior to intervention for CTO lesions to maintain an activated clotting time of ≥300 s.

### Hemostasis and Follow-Up

After the procedure, a small pile of gauze was placed over the puncture site during sheath removal, followed by the application of a semi-elastic bandage, which was left *in situ* for 2–3 h. The radial pulse at the distal forearm and in the anatomical snuffbox was checked by manual palpation and Doppler ultrasound before discharge and at the 3-month follow-up period. After PCI, the patients received dual antiplatelet therapy for 1 year.

### Statistical Analysis

Data are presented as means ± standard deviation and numbers with percentages. The characteristics of the study groups were compared using the *t*-test for continuous variables and the Chi-square-test for categorical variables. Statistical analysis was performed using the statistical software program SPSS (version 22.0; SPSS Inc., Chicago, Illinois, USA) A probability value of <0.05 was considered statistically significant.

## Results

### Baseline Characteristics

The demographic data are presented in Table 1. The mean age of the patients was 61 ± 11 years, and 266 patients (89.3%) were men. Among the study population, 31 patients (11.4%) had left main artery disease, and 34 (10.4%) had more than 2 CTO lesions. The mean left ventricular ejection fraction was 55.0 ± 11.1%. Most indications for CTO intervention were stable angina (54.4%) and acute coronary syndrome (estimated at 39.3%). A total of 262 patients (87.9%) had prior puncture of the radial artery. The mean duration of hospitalization was 5 ± 3 days.

**Table 1:** Demographics and clinical characteristics.

**Variables**	**All patients (*N* = 298)**
**Demographic**	
Age (years)	61 ± 11
Male gender (%)	261 (87.3)
Body height (cm)	166.3 ± 8.7
Body weight (kg)	74.5 ± 13.3
**Medical history**	
Diabetes mellitus (%)	141 (47.3)
Hypertension (%)	266 (89.3)
Dyslipidemia (%)	257 (86.5)
Prior stroke (%)	22 (7.4)
Previous MI (%)	117 (39.4)
PVD (%)	11 (3.7)
ESRD (%)	7 (2.3)
History of CABG (%)	7 (2.3)
Smoker (%)	159 (53.5)
**Coronary artery disease**	
Left main disease (%)	34 (11.4)
≥ two CTO lesions (%)	31 (10.4)
**LVEF (%)**	55.0 ± 11.1
**Lab data**	
Creatinine (excluded ESRD) (mg/dL)	1.06 ± 0.36
LDL (mg/dL)	117.4 ± 37.3
**Reason for CTO PCI**	
Stable angina (%)	162 (54.4)
Unstable angina (%)	83 (27.9)
Recent acute MI or NSTEMI (%)	34 (11.4)
Heart failure (%)	19 (6.4)
**Prior puncture at conventionally radial site**	262 (87.9)
**Total hospitalization day (days)**	5 ± 3

*Data are expressed as mean ± standard deviation or as a number (percentage)*.

*N, number; MI, myocardial infarction; PVD, peripheral vascular disease; ESRD, end-stage renal disease; CABG, coronary artery bypass surgery; CTO, chronic total occlusion; LVEF, left ventricular ejection fraction; LDL, Low-density lipoprotein cholesterol; PCI, percutaneous coronary intervention; NSTEMI, non-ST-segment elevation myocardial infarction*.

### Procedural Characteristics of the Snuffbox Approach

The procedural characteristics and success rate of dTRA are listed in Table 2. The average puncture time was 4.57 ± 2.91 min for 419 punctures. The overall success rate of snuffbox access was 96.9% (406/419), 96.5% (276/286) in the group with left snuffbox access, and 97.7% (130/133) in the group with right snuffbox access. Bilateral snuffbox access was performed in 107 patients (35.9%). If physician wanted to perform contralateral injection for retrograde approach, we attempted bilateral dTRA first. A Glidesheath was used in 400 dTRA (95.5%) for CTO intervention. Among these, 6-F Glidesheath was inserted in 81.5% access sites, and 7-F Glidesheath was inserted in 18.5% access sites.

**Table 2:** Procedural characteristic of the snuffbox approach.

**Variable**	**(*N* = 298; *P* = 419)**
**Access time (minutes)**	4.57 ± 2.91
**Different puncture sites**	
Success rate of all snuffbox puncture (%)	96.9 (406/419)
Success rate of left snuffbox puncture (%)	96.5 (276/286)
Success rate of right snuffbox puncture (%)	97.7 (130/133)
**Bilateral snuffbox access (%)**	107 (35.9)
**The use of Glidesheath (%)**	400 (95.5)

*Data are expressed as mean ± standard deviation or as a number (percentage)*.

*N, number; P, puncture*.

### Procedural Characteristics and Characteristics of the CTO Lesions

Table 3 displays the characteristics of 307 CTO lesions. Chronic total occlusion lesions were located at the right coronary artery (RCA) (50.5%), left anterior descending artery (LAD) (39.4%), left circumflex artery (10.1%). The mean J-CTO (Japanese chronic total occlusion) score was 2.6 ± 0.9 points, and the J-CTO score of 159 (51.8%) CTO lesions was ≥3 points. The mean length of the CTO lesions was 24.3 ± 11.2 mm, and the mean total lesion length was 59.4 ± 20.0 mm.

**Table 3:** The characteristics of the CTO lesions and procedural characteristics.

**Variable**	**All patients**	**Antegrade**	**Retrograde**	***P*-value**
	**(*N* = 298;** ***L* = 307)**	**(*N* = 195;** ***L* = 201)**	**(*N* = 103;** ***L* = 106)**	
**CTO characteristics**				
Target CTO location				0.005
RCA	155 (50.5)	88 (43.8)	67 (63.2)	
LAD	121 (39.4)	91 (45.3)	30 (28.3)	
LCX	31 (10.1)	22 (10.9)	9 (8.5)	
J-CTO score	2.6 ± 0.9	2.3 ± 0.9	3.0 ± 0.9	<0.001
J-CTO score (≥3) (%)	159 (51.8)	85 (42.3)	74 (69.8)	<0.001
Heavy calcification (%)	46 (15.0)	26 (12.9)	20 (18.9)	0.181
Bending >45 degree (%)	31 (10.1)	12 (6.0)	19 (17.9)	0.002
Blunt or no stump (%)	254 (82.7)	160 (79.6)	94 (88.7)	0.056
Re-attempt (%)	126 (41.0)	62 (30.8)	64 (60.4)	<0.001
CTO length >20 mm (%)	184 (59.9)	107 (53.2)	77 (72.6)	0.001
Side branch at occluded site (%)	221 (72.0)	145 (72.1)	76 (71.7)	1.000
In-stent restenosis (%)	38 (12.6)	30 (14.9)	8 (7.5)	0.069
CTO lesion length (mm)	24.3 ± 11.2	22.0 ± 8.6	28.6 ± 13.9	<0.001
Total lesion length (mm)	59.4 ± 20.0	55.5 ± 19.3	66.8 ± 19.4	<0.001
Post-PCI stenosis (%)	17.1 ± 7.5	14.7 ± 6.4	21.7 ± 8.3	0.007
Post-PCI MLD (mm)	2.78 ± 1.04	2.79 ± 0.91	2.75 ± 1.26	0.748
Post-PCI RLD (mm)	3.27 ± 0.43	3.20 ± 0.40	3.40 ± 0.47	<0.001
**Procedural characteristics**				
Procedure time (minutes)	140.0 ± 55.6	121.2 ± 50.9	163.9 ± 53.6	<0.001
Fluoroscopy time (minutes)	55.3 ± 25.7	48.0 ± 22.4	69.2 ± 25.9	<0.001
Contrast volume (ml)	289.1 ± 78.6	273.8 ± 78.4	318.1 ± 70.5	<0.001
Contralateral injection (%)	267 (87.0)	163 (81.1)	104 (98.1)	<0.001
**CTO technique**				
Externalization (%)	95 (30.9)	29 (14.4)	66 (62.3)	<0.001
CART or reverse CART (%)	94 (30.6)	28 (13.9)	66 (62.3)	<0.001
Marker or kissing wire (%)	121 (39.4)	36 (17.9)	85 (80.2)	<0.001
Knuckle wire (%)	56 (18.2)	20 (10.0)	36 (34.0)	<0.001
**Special device**				
Rotablater use (%)	9 (2.9)	8 (4.0)	1 (0.9)	0.171
IVUS or OCT guided (%)	270 (87.9)	172 (86.0)	98 (93.3)	0.060
**Wire number**	6 ± 2	5 ± 2	7 ± 2	<0.001
**Technical success rate (%)**	289 (94.1)	194 (96.5)	95 (89.6)	0.015
**Procedural success rate (%)**	287 (93.5)	194 (96.5)	93 (87.7)	0.006
**Procedural complications (%)**				
Cardiac tamponade need pericardiocentesis (%)	7 (2.3)	4 (2.0)	3 (2.8)	0.696
Wire perforation at collateral vessel without tamponade (%)	24 (7.8)	13 (6.5)	11 (10.4)	0.265
Major side branch acute occlusion (%)	28 (9.1)	15 (7.5)	13 (12.3)	0.210
Rotablater incarceration (%)	1 (0.3)	1 (0.5)	0 (0)	1.000
Aortic cuspid dissection (%)	2 (0.7)	1 (0.5)	1 (0.9)	1.000

*Data are expressed as mean ± standard deviation or as a number (percentage)*.

*CTO, chronic total occlusion; LAD, left anterior descending artery; LCX, left circumflex artery; RCA, right coronary artery; J-CTO, Japanese chronic total occlusion; PCI, percutaneous coronary intervention; MLD, minimal luminal diameter; RLD, reference luminal diameter; CART, controlled antegrade and retrograde subintimal tracking; PCI, percutaneous coronary intervention; IVUS, intra-vascular ultrasound; OCT, optical coherence tomography*.

The procedural characteristics are listed in Table 3. The mean procedure time was 140.0 ± 55.6 min, the mean fluoroscopy time was 55.3 ± 25.7 min. The mean contrast volume was 289.1 ± 78.6 ml. Contralateral injection were used in 267 (87.0%) CTO lesions. A total of 195 patients (201 lesions) received antegrade attempt, and 103 patients (106 lesions) received retrograde attempt. Externalization was performed in 95 (30.9%) lesions, and CART or reverse-CART was performed in 94 (30.6%) lesions. The marker or Kissing wire technique was used in 121 (39.4%) lesions, and Knuckle wire was used in 56 (18.2%) lesions. Nine (2.9%) lesions required the use of a rotablator for debulking. Intra-vascular ultrasound or optical coherence tomography-guided PCI was performed in 270 (87.9%) lesions. The technical success rate was 94.1%, and the procedural success rate was 93.5%. Seven patients (2.3%) suffered from cardiac tamponade related to wire perforation and required pericardiocentesis. Other procedural complications included wire perforation without tamponade (24 patients−7.8%), major side branch occlusion (28 patients−9.1%), rotablator incarceration (one patient−0.3%), and aortic cuspid dissection (two patients−0.7%).

The sites of CTO lesions were significantly different (*p* = 0.005) between the antegrade and retrograde approaches; most lesions in the antegrade approach were at the LAD, and most lesions in the retrograde approach were at the RCA. The mean J-CTO score was significantly different between the two groups; higher J-CTO scores were noted in the population with the retrograde approach (antegrade vs. retrograde, 2.3 ± 0.9 vs. 3.0 ± 0.9; *p* < 0.001). Furthermore, a higher percentage of reattempt lesions (antegrade vs. retrograde, 30.8 vs. 60.4%; *p* < 0.001) and a higher percentage of CTO length > 20 mm (antegrade vs. retrograde, 53.2 vs. 72.6%; *p* = 0.001) were noted in the population with the retrograde approach. In addition, longer CTO lesions and total lesions, longer procedure time, longer fluoroscopy time, and increased contrast volume were noted in the population with the retrograde approach. The technical success rate (antegrade vs. retrograde, 96.5 vs. 89.6%; *p* < 0.001) and procedure success rate (antegrade vs. retrograde, 96.5 vs. 87.7%; *p* < 0.001) differed significantly between the groups, while the incidence of procedural complications did not.

### Access-Site Vascular and Procedural Complications and In-Hospital Outcomes

Nine patients (2.1%) experienced mild ecchymosis. Severe access-site vascular complications occurred in two patients. One patient (0.1%) experienced severe forearm hematoma due to the perforation of a small branch of the brachial artery and required blood transfusion. One patient (0.1%) presented with iatrogenic arteriovenous fistula formation, which was noted 3 months later and spontaneously healed 9 months later. No patient experienced spasm during puncture and two patients (0.5%) suffered from distal radial artery occlusion during follow-up period.

Fourteen patients (4.6%) had periprocedural MI, and one patient (0.3%) experienced post-PCI acute kidney injury (Table 4). Five patients (1.7%) experienced acute or subacute instent thrombosis. Two patients (0.7%) experienced in-hospital complications. One patient (0.3%) experienced non-fatal MI due to acute in-stent thrombosis. One patient (0.3%) experienced wire perforation-related cardiac tamponade and died because of severe heart failure.

**Table 4:** Access-site vascular and procedural complications and in-hospital outcomes.

**Variables**	**Value (*N* = 298; *P* = 419)**
**Access-site vascular complications**	
Mild ecchymosis (%)	9 (2.1)
Hematomas need blood transfusion (%)	1 (0.2)
Arteriovenous fistula (%)	1 (0.2)
Radial artery occlusion (%)	2 (0.5)
**Procedural complications**	
Peri-procedure infarction (%)	14 (4.6)
Post-PCI AKI (%)	1 (0.3)
Acute or subacute instent thrombosis (%)	5 (1.7)
**In-hospital outcomes**	
**Total MACCE (%)**	2 (0.7)
Non-fatal MI (%)	1 (0.3)
Stroke (%)	0 (0)
CV mortality (%)	1 (0.3)
**All-cause mortality (%)**	1 (0.3)

*Data are expressed as a number (percentage)*.

*N, number; P, puncture; MACCE, major adverse cardiovascular and cerebral events; MI, myocardial infarction; CV, cardiovascular*.

## Discussion

To the best of our knowledge, this is the first study in which the snuffbox CTO registry was used to assess the feasibility of CTO-PCI through dTRA. A high procedural success rate was achieved (antegrade: 96.5%; retrograde: 87.7%), with a low rate of severe access site complications (0.5%). Moreover, low incidence of distal radial artery occlusion was noted. Accordingly, dTRA in the anatomical snuffbox for CTO intervention is both applicable and safe. Moreover, we could perform angioplasty for radial artery occlusion first via dTRA if ipsilateral radial artery occlusion.

### Applications and Limitations of the TRA CTO-PCI

The key point of the CTO PCI procedure is the effective backup support from larger guiding catheters. Although, conventional 6-F guide catheter systems may offer enough working space for complex devices or PCI techniques, they are less useful for CTO-PCI. Therefore, 7-F guiding catheters are frequently exchanged during TRA CTO-PCI (13). However, the most critical limitation of TRA-PCI is the relatively small size of the radial artery in comparison to the femoral artery. Indeed, in a Japanese population, a 7-F conventional sheath could be physically inserted via the radial artery in 71.5% of male patients and in only 40.3% of female patients (13). During clinical follow-up, a 6–11% occlusion rate was reported with a 6-F artery sheath system, while a 1–3% occlusion rate was reported with a 5-F system (14). We previous demonstrated that although a conventional 7-F artery sheath could be inserted via high radial artery sites with only 3.5% forearm ecchymoses of access sites, puncture via a high radial artery site was more difficult than via the usual radial artery site; it also required a learning curve (15). After the procedure, the incidence of radial artery occlusion ranged from <1 to 33% and varied with the timing of the assessment of radial artery patency (9). On the other hand, we still could perform PCI via dTRA if ipsilateral radial artery occlusion. In our study, 262 patients (87.9%) received prior PCI at conventionally radial site and two patients presented ipsilateral radial artery occlusion. We performed angioplasty for ipsilateral radial artery occlusion first via dTRA.

### Routine Utilization of dTRA Access for CTO PCI

Kiemeneij et al. reported that dTRA is safe and feasible for coronary angiography and non-complex PCI, and has many advantages to both, the patient and the operator (11). However, the most critical limitation of dTRA PCI is the relatively small size of the distal radial artery in comparison to the standard radial artery. Thin-walled sheaths (Glidesheath) can be effectively used for TRA-PCI, as the 7-F Glidesheath has a similar diameter to the Standard 6-F sheath, which is suitable for a smaller distal radial artery and more convenient for complex intervention.

In the current study, only patients who received the dTRA approach for CTO lesions were enrolled. A 6-F (81.5%) or 7-F Glidesheath (18.5%) was used in 400 dTRA sites. Multiple complex procedures were successfully performed, such as the CART or reverse-CART technique, externalization, thrombectomy, IVUS-guided PCI, kissing or anchoring balloon techniques, rotational atherectomy, and even deployment of the Cover stent for coronary perforation. Moreover, we successfully downsized the conventional arterial sheath from a 7-F (transfemoral PCI), 6-F (TRA-PCI), and a sheathless guiding catheter system. We only failed to obtain left snuffbox access in 10 patients (2.3%) and right snuffbox access in 3 patients (3.5%) and switched to conventional TRA or opposite dTRA in these cases. The mean access time of dTRA was 4.57 ± 2.91 min. Two patients developed severe access site-related complications, one developed arteriovenous fistula at 3 months follow-up and healed spontaneously 9 months later, and the other developed forearm ecchymosis due to small branch perforation during advancement of the Terumo 0.025 wire.

Kim et al. reported the Doppler ultrasound results of a patient receiving PCI via the left snuffbox approach and showed that the maximal diameter of the snuffbox radial artery was significantly smaller than that of the conventional RA (2.57 vs. 2.72 mm) (16). The outer diameter of the 7-F Glidesheath is 2.79 mm, and that of the 6-F is 2.46 mm. In our study, 95.5% of the dTRA received a Glidesheath (6-F: 81.5%; 7-F: 18.5%) for CTO intervention. Furthermore, no patient suffered from severe radial artery spasm that would have aborted the procedure and only two patients developed distal radial artery occlusion. Our study demonstrated a high procedural success rate up to 93.5% (antegrade: 96.5%; retrograde: 87.7%). Compared to our previous study, the procedural success rate of TRA was 87.1% (17), and from 81.7 to 88.2% in the population using the retrograde approach (18). Therefore, dTRA plus Glidesheath may not influence the success rate of CTO intervention.

### Access-Site Vascular Complications After dTRA

In our study, only two severe access-site vascular complications (ASVC) were recorded during dTRA CTO-PCI; these two cases were categorized as “very early” and “early” diagnoses of ASVC. None of the patients required surgery in our study. Burzotta et al. concluded that prompt recognition is pivotal as a “late” diagnosis which is associated with the need for surgery (19). Compared with the standard arterial sheath technique, dTRA access was not associated with higher ASVC in our study.

### Limitations

Our study has several important limitations to our study. First, this was a retrospective study and from a single medical center for selective patients, and we did not randomize the patients to compare dTRA with conventional TRA. Second, the CTO procedures were performed by one major operator. Third, the data on success and failure reported by operators and not blinded physicians. Fourth, we used manual palpation and Doppler ultrasound to check the post-PCI patent rate of distal radial arteries but did not record detailed information about stenosis and vessel diameter. However, we still provide particularly important information about the dTRA approach for complex CTO intervention. A large-randomized study is required to compare the results between dTRA and conventional TRA for CTO intervention.

## Conclusions

It is both safe and feasible to use dTRA plus Glidesheath for complex CTO intervention. The incidences of procedure-related complications and severe access-site vascular complications, and distal radial artery occlusion were low.

## Data Availability Statement

The raw data supporting the conclusions of this article will be made available by the authors, without undue reservation.

## Author Contributions

C-JL and C-JW contributed to the conception, design of the study, and organized the database. W-CL performed the statistical analysis. C-JL wrote the first draft of the manuscript. W-CL, C-HL, W-JC, S-KH, C-JC, C-HY, H-YF, and C-IC wrote sections of the manuscript. All authors contributed to manuscript revision, read, and approved the submitted version.

## Conflict of Interest

The authors declare that the research was conducted in the absence of any commercial or financial relationships that could be construed as a potential conflict of interest.

## Supplementary Material

Supplementary Figure 1 Demonstration of the bilateral snuffbox approach. A 7-F Glidesheath via the left snuffbox and a 7-F standard sheath via the right snuffbox.
