# Supplementary figures and images for: Feasibility and Safety of Chronic Total Occlusion Percutaneous Coronary Intervention via Distal Transradial Access
Source: Front Cardiovasc Med. 2021 May 10;8:673858. doi: 10.3389/fcvm.2021.673858 (PMC8141614; doi:10.3389/fcvm.2021.673858)

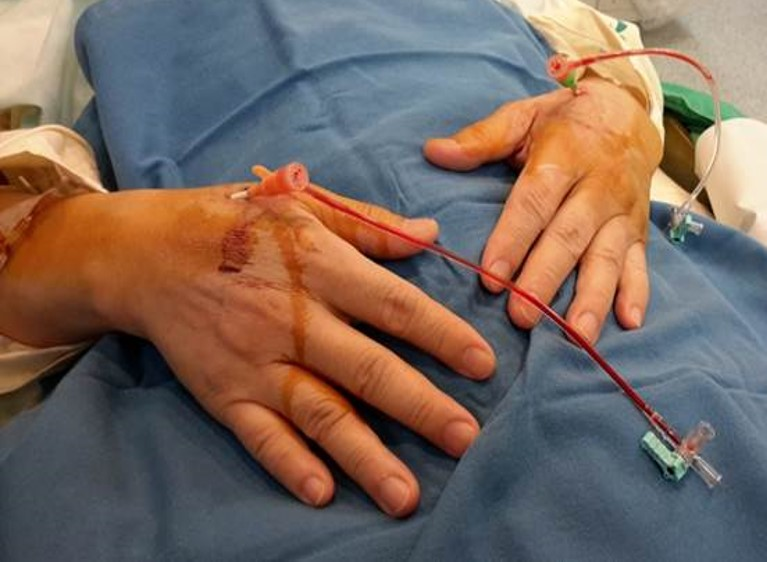

Supplement: Supplementary Figure 1 — Demonstration of the bilateral snuffbox approach. A 7-F Glidesheath via the left snuffbox and a 7-F standard sheath via the right snuffbox. [file Image_1.TIF]
